# Society Objects

**Published:** 1887-10

**Authors:** 


### SOCIETY OBJECTS.

The supposed object for which all dental societies exist is professional improvement, and unless they accomplish all that is within their power for the good of dentistry the members are certainly derelict to duty. Without a literature there can be no profession, for the very word presupposes knowledge generally diffused. It is the duty then, of every member who loves his profession, to do what he can for this spread of information. The society which keeps its knowledge confined within its own society circles, or which uses it to build up and sustain any selfish end or object, has no more of real professional spirit than had the old exclusive practitioners, who, in early days, kept their laboratory doors locked lest their brother might profit by association with them.

